# Correction to: Downregulated exosomal microRNA-148b-3p in cancer associated fibroblasts enhance chemosensitivity of bladder cancer cells by downregulating the Wnt/β-catenin pathway and upregulating PTEN

**DOI:** 10.1007/s13402-021-00615-y

**Published:** 2021-07-20

**Authors:** Guang Shan, Xike Zhou, Juan Gu, Daoping Zhou, Wei Cheng, Huaiguo Wu, Yueping Wang, Tian Tang, Xuedong Wang

**Affiliations:** 1 Department of Urology, RenMin Hospital of Wuhan University, Wuhan, 430060 Hubei People’s Republic of China; 2 Department of Medical Laboratory Science, The Fifth People’s Hospital of Wuxi, Nanjing Medical University, 1215 Guangrui Road, Jiangsu 214000 Wuxi, People’s Republic of China; 3 Department of Pathology, The Fifth People’s Hospital of Wuxi, The Medical School of Jiangnan University, Jiangsu 214000 Wuxi, People’s Republic of China; 4 Center for Precision Medicine, Anhui No.2 Provincial People’s Hospital, Hefei, 230041 Anhui People’s Republic of China; 5 Department of Biology, College of Arts & Science, Massachusetts University, Boston, MA 02125 USA; 6 Department of Oncology, RenMin Hospital of Wuhan University, Hubei 430060 Wuhan, People’s Republic of China

**Correction: Cellular Oncology (2021) 44:45–59**

10.1007/s13402-020-00500-0

The affiliations of the 1st, 7th and 9th authors are revised.

The original article has been corrected.

